# Prediction of Breast Cancer Survival Through Knowledge Discovery in Databases

**DOI:** 10.5539/gjhs.v7n4p392

**Published:** 2015-01-25

**Authors:** Hadi Lotfnezhad Afshar, Maryam Ahmadi, Masoud Roudbari, Farahnaz Sadoughi

**Affiliations:** 1 Department of Health Information Management, School of Health Management and Information Sciences, Iran University of Medical Sciences, Tehran, Iran; 2 Health Management and Economics Research Center, School of Health Management and Information Sciences, Iran University of Medical Sciences, Tehran, Iran; 3 Department of Biostatistics, School of Public Health, Iran University of Medical Sciences, Tehran, Iran

**Keywords:** breast neoplasms, survival, data mining

## Abstract

The collection of large volumes of medical data has offered an opportunity to develop prediction models for survival by the medical research community. Medical researchers who seek to discover and extract hidden patterns and relationships among large number of variables use knowledge discovery in databases (KDD) to predict the outcome of a disease. The study was conducted to develop predictive models and discover relationships between certain predictor variables and survival in the context of breast cancer. This study is Cross sectional. After data preparation, data of 22,763 female patients, mean age 59.4 years, stored in the Surveillance Epidemiology and End Results (SEER) breast cancer dataset were analyzed anonymously. IBM SPSS Statistics 16, Access 2003 and Excel 2003 were used in the data preparation and IBM SPSS Modeler 14.2 was used in the model design. Support Vector Machine (SVM) model outperformed other models in the prediction of breast cancer survival. Analysis showed SVM model detected ten important predictor variables contributing mostly to prediction of breast cancer survival. Among important variables, behavior of tumor as the most important variable and stage of malignancy as the least important variable were identified. In current study, applying of the knowledge discovery method in the breast cancer dataset predicted the survival condition of breast cancer patients with high confidence and identified the most important variables participating in breast cancer survival.

## 1. Introduction

Breast cancer is the most common malignancy among women that causes large number of neoplastic deaths across worldwide. It is the fifth cause of death due to malignancies among Iranian women with approximately 8500 incident cases per year (Hadi, Asadollahi, & Talei, 2009; Movahedi et al., 2012). Once a patient is diagnosed with breast cancer, the malignant lump must be excised. During this procedure, physicians must determine the prognosis of the disease. This is the prediction of the expected flow of the disease. Prognosis is important because the type and intensity of the medications are based on it (Gupta, Kumar, & Sharma, 2011). Survival analysis is a field in medical prognosis that deals with application of various methods to data stored in health datasets in order to predict the survival of a particular patient suffering from a disease over a particular time period (Delen, Walker, & Kadam, 2005). The collection of large volumes of health data has offered an opportunity to develop prediction models for survival by the health research community. Health researchers who seek to discover and extract hidden patterns and relationships among large number of variables use knowledge discovery in databases (KDD) to predict the outcome of a disease (Bellazzi et al., 2011; Cios & William Moore, 2002). KDD as a process consists of an iterative sequence of the following steps: understanding the domain of research field (i.e., health domain), understanding the data used in domain, handle missing values and remove irrelevant or redundant variables (data preparation), applying methods in order to extract data patterns (data mining), and knowledge presentation (Delen et al., 2005; Han, Kamber, & Pei, 2011).

The extraction of pattern representing survival status of patients with breast cancer from demographic and clinical data is the main object of KDD in the health domain (Cruz & Wishart, 2007; Jerez et al., 2005). Data mining technique is a part of KDD process that according to the discovered pattern can predict whether a new patient will survive from a disease such as breast cancer within a particular time period (Razavi, Gill, Åhlfeldt, & Shahsavar, 2007).

Predicting survival condition of breast cancer patients by considering their risk factors is difficult. The abnormal values of some morphological and pathological tumor specifications and biological tumor markers are known as risk factors. Choosing the most appropriate treatment for the patients and assign those to high-risk groups are related to identification of risk factors that increase the mortality of cancer. The role of domain experts in predicting breast cancer survival with respect to important risk factors is undeniable. However, the availability of these experienced oncologists is limited. The support of less experienced oncologists with expert knowledge in order to care for their patients is a considerable challenge (Fieschi, Dufour, Staccini, Gouvernet, & Bouhaddou, 2003). In these circumstances, using the hidden experiences stored in electronic or paper records to support less experienced physicians in their daily decision-making is an effective solution (Windle, 2004). Applying KDD process generally and data mining methods particularly as decision support systems (DSS) to predict the survival of new patients is a great advantage and new field for health researchers studying the relationships between risk factors and survival of cancers (Lee, Williams, & Cheon, 2008).

Delen and et al used a large breast cancer dataset and applied KDD to develop DSS for breast cancer survival. Their study showed the high potential of KDD process in accurate prediction of breast cancer survival (Delen et al., 2005). Jerez and et al analyzed data of high risk breast cancer patients with different approach of KDD and traditional statistical method. The performance of KDD process was better than statistical method in prognosis analysis of breast cancer (Jerez et al., 2005). Razavi and et al compared performance of KDD process and domain experts in prognosis of breast cancer. Their result showed that performance of KDD was better than domain experts (Razavi et al., 2007). Thongkam and et al stated that for reaching to the highest performance of KDD process in breast cancer prognosis, data preparation step should be done with high quality and large data (Thongkam, Xu, Zhang, & Huang, 2009).

The purpose of this study is to develop predictive models and discover relationships between certain predictor variables and survival in the context of breast cancer.

## 2. Method

### 2.1 Data Source

This study is Cross sectional. In this research, the Surveillance Epidemiology and End Results (SEER) breast cancer dataset was used. This study is a Cross sectional and the required data were obtained from the Surveillance Epidemiology and End Results (SEER) breast cancer dataset. This dataset contains 657,712 records and 72 variables. These variables provide socio-demographic and cancer specific information. Each record represents a particular patient within the database. In this study follow-up patients by 2009 that were diagnosed as breast cancer from 1999 to 2004 were selected. The records of patients diagnosed with breast cancer between 1999 and 2004 were selected. They had been followed for 5-years.

### 2.2 Data Preparation

In order to build the best possible predictive model, the following steps were performed as data preparation:

After studying the data dictionary of dataset, the variables of unrelated to breast cancer were removed.

The integrated variables were separated into the different variables. For example, variables: *Histology*, *Behavior* and *Grade code* that are important variables to predict breast cancer survival were a part of *Morphology* variable. Also, aggregated *Extent of Disease* variable was separated into the six different tumor attributes. The variables integrated into a general variable were disintegrated because they contained distinct information about cancer. For example, variables: Histology, Behavior and Grade code that are important variables to predict breast cancer survival were a part of Morphology variable. Also, aggregated Extent of Disease variable was separated into the six different tumor attributes.

For extracting records between1999 and 2004, the dataset was exported from IBM SPSS Statistics 16 to Access 2003 and 22,763 records were obtained.

They were evaluated to determine inaccuracy, inconsistency and missingness in data. For instance, early evaluation demonstrated that 55 percent of variables: *Tumor Size*, *Extension* and *Lymph node involvement* had missing values, but reviewing of data dictionary showed that they only had been registered for years between 1998 and 2003. For records related after 2003, variables: *Collaborative Stage (CS) Tumor Size*, *Collaborative Stage (CS) Extension* and *Collaborative Stage (CS) Lymph node involvement* were available and had 45, 0 and 45 percent missing values respectively. Some missing values of six old variables were in new variables and vice versa. To solve this problem, a simple mapping was done in Excel 2003 and these variables were converted to three ones. Table 1 shows frequency and percentage of dataset missing values.

**Table 1:** The missing values of predictor variables

Variables	Frequency	Percentage
Race	85	0.5
Marital status	674	3.8
Primary site code	4196	23.8
Histology	0	0
Behavior	0	0
Grade	3033	17.2
Extension of tumor	384	2.2
Lymph node involvement	647	3.7
Radiation	131	0.7
Stage	388	2.2
Site specific surgery code	68	0.4
ERStatus	0	0
PRStatus	0	0
Age	0	0
Tumor size	2930	17.9
Number of positive nodes	102	0.6
Number of nodes	96	0.5
Number of primaries	0	0

The review of published papers and counseling consulting with oncologists were performed to determine of predictor variables for survival modeling (Table 2) (Amna, Umer, Ali, & Minkoo, 2010; Bellaachia & Guven, 2006; Burke et al., 1997; Delen et al., 2005; A Endo, Shibata, & Tanaka, 2008; Arihito Endo, Takeo, & Tanaka, 2007).

**Table 2:** Predictor variables

*Categorical variables*	*Number of distinct values*		
Race	18		
Marital status	5		
Primary site code	8		
Histology	55		
Behavior	2		
Grade	4		
Extension of tumor	8		
Lymph node involvement	9		
Radiation	8		
Stage	4		
Site specific surgery code	40		
ERStatus	4		
PRStatus	4		
*Continuous variables*	*Mean*	SD*	*Range*

Age	59.4	13.5	17-103
Tumor size	18.3	18.2	0-555
Number of positive nodes	1.1	3	0-45
Number of nodes	6.2	7.6	0-90
Number of primaries	1.3	0.6	1-6

* Standard deviation.

Dependent variable was created by the method introduced in by Bellaachia paper (Bellaachia & Guven, 2006). This variable is a binary one that 1 and 0 are representatives of *death* and *aliveness* respectively. The percentages of *death* and *aliveness* values are were 10.3 and 89.7 respectively.

For handling missing values, multiple imputation (MI) method was used in the IBM SPSS Statistics 16. This method analyzes the patterns of missing values and then produces the multiple versions of the dataset that each contains its own set of imputed values. When running the analysis on each complete dataset, results of all datasets are averaged and a single one is produced. For MI, the pattern of data must be missing at random (Arbuckle, 2011; Liu Peng, 2005).

The values of dependent variable were not balanced (*aliveness* values were approximately nine times greater than *death* values). In these situations, the results of data mining are biased towards the majority value. For solving this problem, under-sampling or over-sampling is used. Under-sampling is used to decrease the size of the majority value to the same size of the minority value, whereas over-sampling is used to increase the size of the minority value to the same size of the majority value. The over-sampling method was used to increase *dead* values to the same numbers of *aliveness* values.

### 2.3 Data Mining

For applying data mining step, IBM SPSS Modeler 14.2 was used. The 70 (15934) and 30 (6829) percent of database records were selected as training and testing data respectively. Training data are used to construct or discover a predictive model and testing data are used to evaluate performance of model (Thongkam et al., 2009; Witten, Frank, & Hall, 2011).

Three different types of methods: Support Vector Machine (SVM), Bayes Net, and CHi-squared Automatic Interaction Detection (CHAID) were used as prediction models. SVM is one of the supervised learning algorithms with well-built regularization properties. The optimization procedure of SVM maximizes predictive accuracy and also reduces the overfitting (more accurate in fitting known data but less accurate in predicting new data) of the training data. Basically SVM spins around the idea of finding optimal decision boundary i.e. maximizing the margin by finding the largest achievable distance among the separating hyperplane and the instances on either side of it (Amna et al., 2010). The Bayes Network is capable of learning the probability density functions of individual pattern classes from a collection of learning samples, designed for pattern classification based on the Bayesian decision rule (A Endo et al., 2008). CHAID is a type of decision tree technique, based upon adjusted significance testing. CHAID can be used for prediction as well as classification, and for detection of interaction between variables (Han et al., 2011).

Accuracy, sensitivity, specificity and adjusted propensity were used for measuring the prediction models performances. Accuracy is the percentage of testing data that are correctly predicted by the model. Sensitivity and specificity are also calculated from the accuracy (Han et al., 2011). In breast cancer field, sensitivity is the proportion of breast cancer patients and specificity is the proportion of non-breast cancer patients that are correctly identified by the model. The adjusted propensity is a measure of how ‘confident’ the model is in its prediction and should be used alongside other measurements particularly in the unbalanced dependent variable (Reynolds et al., 2009).

## 3. Results

SVM outperformed other models in the prediction of breast cancer survival. This superiority was in all of the measurement criteria. Table 3 shows the results.

**Table 3:** The comparison of data mining models in the prediction of breast cancer survival

	Sensitivity	Specificity	Accuracy	Adjusted Propensity scores
SVM	97.7%	95.6%	96.7 %	0.977
Bayes Net	81.8%	86.1%	83.9%	0.880
CHAID	82.2%	82.7%	82.4%	0.829

### 3.1 Identified Variables

**Figure 1:**
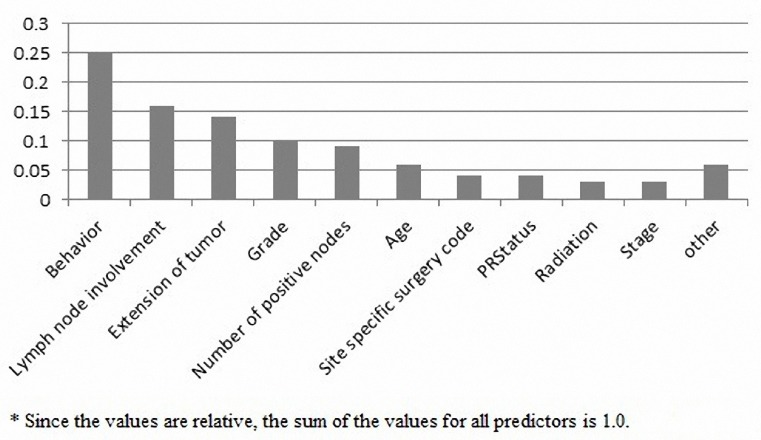
The relative importance of predictor variables identified by SVM in predicting the breast cancer survival^*^

The predictor variables: Behavior, Lymph node involvement, Extension of tumor, Grade, Number of positive nodes, Age, Site specific surgery code, PRStatus, Radiation and Stage contribute mostly to prediction of breast cancer survival in the SVM model. The relative importance of these variables has been showed in Figure 1.

Based on above figure, 25 percent of model predictive power relates to Behavior variable and 6 percent to other variables (least important variables). The relative importance of some predictor variables (Grade, Histology, PRStatus, Lymph node involvement, Site specific surgery code, ERStatus, Race, Marital status, Number of nodes and Stage) identified by the Bayes Net model were almost the same. The CHAID model for predicting breast cancer survival determined the following predictor variables (they are in descending order based on their relative importance): Extension of tumor, Number of positive nodes, Number of nodes, Tumor size, Behavior, ERStatus, PRStatus, Marital status, Age and Grade.

## 4. Discussion

The highest performance of accuracy in our study is 96.7% that belongs to the SVM model. The SVM revealed the highest performance (96.7%) of the accuracy among other models. In the previous studies (Bellaachia & Guven, 2006; Burke et al., 1997; Delen et al., 2005; A Endo et al., 2008; Arihito Endo et al., 2007) that the prediction of breast cancer survival had been performed in the SEER dataset the data miners had not used SVM. Among the used models, the logistic regression had the highest accuracy (85.8%) in the Endo and his colleagues’ (A Endo et al., 2008; Arihito Endo et al., 2007) work; Delen (Delen et al., 2005) and Bellaachia (Bellaachia & Guven, 2006) reported the C5 as the best model in accuracy (93.6% and 86.7% respectively). The Burke and his colleagues had used only the artificial neural network and acquired accuracy was 73%. Because of the differences such as: used software differences in software type, version of dataset, the method of missing values handling and the distribution of dependent variable, the comparison of previous studies results with current study result should be taken into consideration. the comparison of previous studies results with current study result is difficult. However, accuracy in our study in comparison to mentioned studies was better.

Specificity of SVM (95.6%) also is higher than other models. In the medical domain, predicting negative cases (i.e. not survived breast cancer patients) with high accuracy is more important than positive cases (i.e. survived breast cancer patients) (Razavi et al., 2007). In other words specificity is more important than sensitivity. The largest amount of specificity in the Endo (A Endo et al., 2008) and Delen (Delen et al., 2005) works are: 50.9% (artificial neural network) and 91.1% (C5). Specificity had not been reported in the Bellaachia (Bellaachia & Guven, 2006) and Burke (Burke et al., 1997) papers.

The key difference advantage between current study and other studies is reporting adjusted propensity scores. The adjusted propensity score of SVM was better than other models. It predicted breast cancer survival with higher confidence than Bayes Net and CHAID. Balancing of dependent variable in the current study has differentiated it with the previous researches and has necessitated the report of adjusted propensity as the most important criterion in the comparison of models performance. Another advantage of current study is in the way of missing values management. In contrast to this research, the missing values have been deleted in the related studies (Bellaachia & Guven, 2006; Burke et al., 1997; Delen et al., 2005; A Endo et al., 2008;Arihito Endo et al., 2007). The deletion of missing values leads to loss of valuable information and decreases the overall accuracy of models (Magnani, 2004).

In our study, Behavior was the most important variable (25% of relative importance) identified by the best model of this work. This variable in the Bellaachia (Bellaachia & Guven, 2006) and Delen (Delen et al., 2005) studies got 9^th^ (3%) and 10^th^ (9%) rank among other variables. Likely the used model and balancing outcome variable have caused this inconsistency with those studies. Behavior variable determines the general condition of breast cancer (benign, uncertain, in situ and malignant) (Fritz et al., 2000). Among the variables that their relative importance is greater than or equal to 10% in current study, Lymph node involvement and Grade variables are same in the Bellaachia (Bellaachia & Guven, 2006) and Delen (Delen et al., 2005) studies respectively. Lymph node involvement and Grade variables were consistent with Delen (Delen et al., 2005) and Bellaachia (Bellaachia & Guven, 2006) studies. In the former study both variables and in the latter one only Grade variable have been reported.

However in this study the models as artificial neural network, logistic regression and C5 were not used. The other missing values handling methods were not used in this study and a part of the SEER dataset was used other than complete dataset. The mentioned limitations can restrict the findings of this work for generalizing beyond the study conditions.

## 5. Conclusion

In current study, applying of the knowledge discovery method in the breast cancer dataset predicted the survival condition of breast cancer patients with high confidence and identified the most important variables participating in breast cancer survival.
